# Recent advances in understanding ichthyosis pathogenesis

**DOI:** 10.12688/f1000research.8584.1

**Published:** 2016-06-24

**Authors:** Nareh V. Marukian, Keith A. Choate

**Affiliations:** 1 Department of Dermatology, Yale University School of Medicine, New Haven, CT, 06511, USA; 2 Department of Genetics, Yale University School of Medicine, New Haven, CT, 06511, USA; 3 Department of Pathology, Yale University School of Medicine, New Haven, CT, 06511, USA

**Keywords:** ichthyosis, keratinization, hyperproliferation, pathogenesis

## Abstract

The ichthyoses, also known as disorders of keratinization (DOK), encompass a heterogeneous group of skin diseases linked by the common finding of abnormal barrier function, which initiates a default compensatory pathway of hyperproliferation, resulting in the characteristic clinical manifestation of localized and/or generalized scaling. Additional cutaneous findings frequently seen in ichthyoses include generalized xerosis, erythroderma, palmoplantar keratoderma, hypohydrosis, and recurrent infections. In 2009, the Ichthyosis Consensus Conference established a classification consensus for DOK based on pathophysiology, clinical manifestations, and mode of inheritance. This nomenclature system divides DOK into two main groups: nonsyndromic forms, with clinical findings limited to the skin, and syndromic forms, with involvement of additional organ systems. Advances in next-generation sequencing technology have allowed for more rapid and cost-effective genetic analysis, leading to the identification of novel, rare mutations that cause DOK, many of which represent phenotypic expansion. This review focuses on new findings in syndromic and nonsyndromic ichthyoses, with emphasis on novel genetic discoveries that provide insight into disease pathogenesis.

## Introduction

The ichthyoses encompass a heterogeneous group of skin diseases linked by the common finding of abnormal barrier function, which leads to increased transepidermal water loss and compensatory hyperproliferation. The unifying phenotypic feature of the ichthyoses is localized and/or generalized scaling. Other clinical manifestations can include erythroderma (confluent red skin), palmoplantar keratoderma (thickening of the palms and soles), hypohydrosis (diminished sweating), and recurrent infections.

Although ichthyoses are primarily inherited disorders with onset at or shortly after birth, rare acquired forms have been reported in the setting of malignancy, nutritional deficiency, and autoimmune or infectious disease. Mutations in over 50 genes have been reported to cause ichthyoses, and these affect a host of cellular functions including DNA repair, lipid biosynthesis, adhesion, and desquamation as well as other pathways ^1^. Despite myriad pathways for pathogenesis, each features disrupted barrier function.

Epidermal barrier function is maintained by a regular pattern of epidermal renewal in which keratinocytes, the primary cell type of the skin, arise from a renewing stem cell pool and undergo a tightly regulated pattern of differentiation as they transit from the innermost stratum basale to the outermost stratum corneum, where they are ultimately sloughed off. This differentiation program is marked by site-specific expression of proteins and, in the suprabasal layers, the production of components necessary for the generation of the lipid barrier.

**Figure 1.**
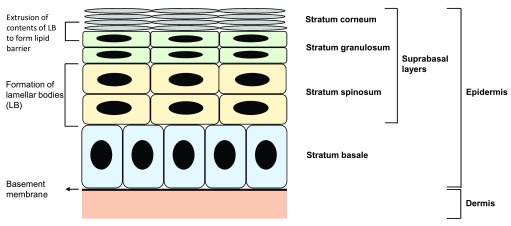
Epidermal structure Epidermal barrier function is maintained by a regular pattern of epidermal differentiation and generation of lipid components.

In the process of differentiation, keratins—intermediate filaments that are responsible for the structural integrity of keratinocytes—are among the first proteins to be expressed in a tightly regulated manner, with keratin 5 and 14 expressed in the basal layer and keratin 1 and 10 expressed in the suprabasal layers. In the stratum spinosum, the second innermost layer of the epidermis, components of the lipid barrier (phospholipids, cholesterol, sphingomyelin, and glucosylceramides) are packaged into lamellar bodies, which are specialized organelles that house the building blocks of the lipid barrier as well as enzymes essential to the processing of lipid barrier precursors. At the transition from the stratum granulosum—the third layer of the epidermis—to the stratum corneum, the contents of lamellar bodies are extruded into the intercellular space to form protective lipid sheets that are responsible for the skin’s hydrophobic barrier ^2, 3^ ( Figure 1).

This overall process of differentiation results in the formation of a robust barrier in the stratum corneum, composed of keratinocytes (the individual bricks of the barrier) and inter-keratinocyte lipids (the mortar) ^4^. Mutations in proteins essential to the formation of this barrier (i.e. keratins and enzymes involved in lipid synthesis) lead to the disruption of barrier integrity, resulting in ichthyosis.

Inherited ichthyoses exhibit marked genetic and phenotypic heterogeneity, and advances in next-generation sequencing technology have allowed for more rapid and cost-effective genetic analysis, leading to the identification of novel, rare mutations that cause DOK. Clear large-scale genotype-phenotype correlations have been difficult to establish, as mutations in the same gene can present with widely divergent phenotypes, even within kindreds bearing the same disease-causing mutation.

In 2009, the Ichthyosis Consensus Conference established a consensus classification for DOK based on pathophysiology, clinical manifestations, and mode of inheritance ^1^. This nomenclature system divides DOK into two main groups: 1) nonsyndromic forms, with clinical findings limited to the skin, and 2) syndromic forms, with involvement of other organ systems.

## Nonsyndromic ichthyoses

### Common ichthyoses

Ichthyosis vulgaris (IV) and X-linked recessive ichthyosis (XLRI) are classified as the “common ichthyoses”, given their high prevalence. IV is the most common form of nonsyndromic inherited ichthyosis, with an estimated incidence of 1 in 250 births ^5^. Typically, IV is a phenotypically mild form of ichthyosis. Clinical findings usually appear at around 2 months of age and include generalized xerosis and fine white to gray scale that is most prominent on the abdomen, chest, and extensor surfaces of the extremities. Keratosis pilaris and hyper-linearity of the palms and soles are also frequently associated with IV.

IV is caused by autosomal dominant mutations in the filaggrin gene ( *FLG*), which plays an essential role in epidermal differentiation and formation of the skin barrier ^6, 7^. An autosomal semidominant mode of inheritance has also been described, meaning that while individuals with heterozygous mutations have a mild phenotype, those with homozygous or compound heterozygous mutations can display more severe forms of ichthyosis ^6^.

Patients with IV are at increased risk for atopic dermatitis, asthma, and allergies ^8, 9^. This increased risk is likely due to disruption of barrier function, which may allow for greater penetration of the epidermis by potential allergens ^8^.

XLRI is the second most common form of inherited ichthyosis, with a prevalence of 1:2000 to 1:6000 in males ^10^. Clinical findings in XLRI are frequently indistinguishable from IV. Manifestations usually first appear in the neonatal period as generalized desquamation and xerosis and progress to fine scaling of the trunk and extremities in infancy. Over time, patients develop brownish, polygonal, plate-like scale that is tightly adherent to the skin. XLRI is caused by mutations in the *STS* gene, encoding steroid sulfatase, on the X chromosome ^11^.

### Autosomal recessive congenital ichthyosis

Autosomal recessive congenital ichthyosis (ARCI) is a genetically and phenotypically heterogeneous group of disorders that includes harlequin ichthyosis (HI), lamellar ichthyosis (LI), and congenital ichthyosiform erythroderma (CIE). The incidence of ARCI has been approximated at 1 in 200,000 births ^12^.

HI is caused by loss-of-function mutations in *ABCA12*, which encodes an ATP-binding cassette (ABC) transporter. *ABCA12* is necessary for lipid transport into lamellar granules and is central to the process of cornification and lipid barrier formation ^13^. Interestingly, while homozygous loss-of-function mutations in *ABCA12* lead to HI, missense mutations in *ABCA12* result in milder phenotypes on the LI/CIE spectrum ^14^. Neonates with HI present with thick, armor-like scale with severe ectropion (eversion of the eyelids), eclabium (eversion of the lips), and flattening of the ears. Some patients with HI die during the neonatal period, but survival has been shown to improve with progress in neonatal intensive care and early treatment with systemic retinoids. Rajpopat *et al.* showed that 83% of HI patients treated with oral retinoids survived compared to 24% of untreated patients ^15^.

LI and CIE represent a spectrum of disorders caused by mutations in one of nine genes: *TGM1*, *NIPAL4/ICHTHYIN*, *ALOX12B*, *ALOXE3*, *CYP4F22*, *ABCA12*, *PNPLA1*, *CERS3,* and *LIPN* ^16^. Mutations in *TGM1* are the most common and account for approximately 32% of heritability of ARCI ^17^. Fisher *et al.* found that mutations in the six most common genes ( *TGM1*, *NIPAL4*, *ALOX12B*, *CYP4F22*, *ALOXE3*, and *ABCA12*) account for 78% of ARCI cases ^17^. Despite this, prior studies of large cohorts of patients with ARCI showed that 22-40% of patients have no mutations in known genes ^17, 18^, highlighting the heterogeneity of this group of disorders and the importance of continued efforts in gene discovery.

### Keratinopathic ichthyosis

Keratinopathic ichthyosis is a group of disorders caused by mutations in the keratin family of genes. The major variant of keratinopathic ichthyosis is epidermolytic ichthyosis (EI). Minor variants include superficial EI (SEI), annular EI (AEI), and ichthyosis Curth-Macklin.

EI is caused by autosomal dominant mutations in the keratin 1 ( *KRT1*) and keratin 10 ( *KRT10*) genes, which play an essential role in maintaining structural integrity in suprabasal keratinocytes ^19^. EI is characterized by marked skin fragility, leading to generalized blister formation on a background of erythroderma. Neonates present with blistering and erythema at birth, but symptoms improve over time. Blistering becomes less frequent and is usually confined to sites of trauma in adulthood. Palmoplantar keratoderma is often associated with EI, although it is more commonly seen in patients with mutations in *KRT1* than *KRT10* ^19^.

SEI, also known as ichthyosis bullosa of Siemens, is caused by mutations in *KRT2* ^20, 21^. Phenotypic manifestations are milder compared to EI and include blister formation in response to trauma and hyperkeratosis (thickening of the stratum corneum) over flexural areas.

AEI is a rare phenotypic variant of EI that was shown by Yang *et al.* to be caused by a unique mutation in *KRT10* that replaces an alanine at residue 12 with a proline ^22^. It is characterized by blister formation at birth, which later progresses to the intermittent development of annular polycyclic erythematous plaques on the trunk and extremities.

Ichthyosis Curth-Macklin is another rare disorder and is caused by autosomal dominant mutations in *KRT1* ^23, 24^. It is characterized by extensive spiky or verrucous hyperkeratosis over the trunk and extensor surfaces of the extremities. It may also be associated with severe palmoplantar keratoderma. In the past 5 years, two novel distinct causative mutations in *KRT1* have been identified in addition to the two mutations that were initially described ^25, 26^. While both EI and ichthyosis Curth-Macklin can be caused by mutations in *KRT1,* EI is caused by amino acid substitutions and in-frame deletions in the gene ^27^, while ichthyosis Curth-Macklin is caused by insertions or deletions that lead to a frameshift ^23– 26^.

## Syndromic ichthyoses

In addition to cutaneous involvement, syndromic ichthyoses affect at least one other organ or system. Many causative genes have been identified for syndromic ichthyoses, including *NSDHL* (CHILD syndrome) ^28^, *EBP* (Conradi-Hunermann-Happle syndrome, CHILD Syndrome) ^28, 29^, and *ALDH3A2* (Sjögren-Larsson syndrome) ^30^. Depending on the specific gene mutated, a wide range of organ systems can be involved, including the skeletal, nervous, endocrine, and cardiovascular systems. Many of the syndromic ichthyoses may present at birth with isolated cutaneous findings, highlighting the importance of a high degree of clinical suspicion and the usefulness of genetic analysis in the early diagnosis of these syndromic cases.

## Recent advances in ichthyosis

### Nonsyndromic ichthyoses

***Mutations in PNPLA1 cause autosomal recessive congenital ichthyosis.*** In 2012, Grall *et al.* reported that mutations in the patatin-like phospholipase domain-containing protein 1 gene ( *PNPLA1*) cause ARCI in Golden Retriever dogs and humans ^31^. Selective inbreeding of dogs to create pure breeds leads to the propagation of not only specific desirable traits but also disease-causing alleles. The selection of these undesirable alleles results in the high prevalence of breed-specific diseases in dogs. For example, the inbreeding of Golden Retrievers has led to the high prevalence of ichthyosis within the breed. The frequency of the mutation in Golden Retrievers is estimated to be approximately 50% ^31^. Ichthyosis in Golden Retrievers presents with generalized scaling, with white or black scale, similar to the phenotypic manifestations of ichthyosis in humans.

Intermarriage within families and breeding approaches for purebred animals provide a unique opportunity to study the genetic basis of rare conditions. Grall *et al.* performed genetic analysis on 20 affected Golden Retrievers, which revealed homozygous mutations in *PNPLA1* in all members of the cohort. Further studies on a human cohort of 46 consanguineous families with ARCI, who were previously found not to have mutations in known ARCI genes, revealed two distinct mutations in *PNPLA1* in two different families ^31^.

The PNPLA family of proteins contains nine members, which play key roles in lipid metabolism ^32, 33^. While disease-causing mutations in other members of the family had been previously identified, mutations in *PNPLA1* had not been previously implicated in any disease ^34– 37^. This finding not only expands the genetic understanding of ARCI but also highlights the essential role of *PNPLA1* in lipid metabolism and maintenance of the barrier function.

***Mutations in *GJA1* cause erythrokeratodermia variabilis et progressiva.*** In 2015, Boyden *et al.* reported that autosomal dominant mutations in *GJA1* cause erythrokeratodermia variabilis et progressiva (EKVP) ^38^, a rare genetic disorder characterized by transient figurate erythematous patches on a background of generalized scaling. *GJA1* encodes connexin 43 (Cx43), which is present throughout the epidermis and is expressed in every tissue type ^39^. Connexins, also known as gap junction proteins, are classified into alpha and beta subgroups ^40^, encoded by *GJA* and *GJB* genes, respectively. Individual connexins form hexamers called connexons.

Connexons can serve two main functions within the plasma membrane—individual connexons can either function as hemichannels, allowing for communication between the cytoplasm and the extracellular space, or dock with connexons on neighboring cells to form gap junctions. Gap junctions are essential to intercellular communication, allowing for synchronization of metabolic and electrical activities between cells as well as the exchange of small molecules and ions. Mutations in connexin genes have been previously shown to cause a wide range of disease phenotypes, including myelin-related disease, skin disease, hearing loss, and congenital cataract ^41^.

While mutations in *GJB3* and *GJB4* have been previously shown to cause EKV/EKVP ^42, 43^, Boyden *et al.* were first to report that mutations in *GJA1* can also cause the phenotype ^38^. Mutations in *GJA1* have been previously described to cause oculodentodigital dysplasia (ODDD) ^39, 44^, which is a systemic disorder with limited cutaneous findings and sharply contrasts with the widespread cutaneous findings with lack of systemic symptoms seen in EKVP. Mutations in *GJA1* that result in EKVP lead to mislocalization of Cx43 ^38^, while mutations resulting in ODDD lead to functionally impaired gap junctions that show a normal pattern of localization ^45^. This finding expands the genetic understanding of EKVP and provides insight into its molecular mechanism.

***Mutations in CARD14 cause pityriasis rubra pilaris.*** Pityriasis rubra pilaris (PRP) is a papulosquamous disorder that is characterized by well-demarcated salmon-colored plaques with fine scale, palmoplantar keratoderma, and follicular hyperkeratosis (excessive accumulation of keratin in hair follicles) that presents shortly after birth or can be acquired later in life, typically in the fourth or fifth decade. Although phenotypic features of PRP overlap with psoriasis, the two can be distinguished based on distinct clinical and histopathological factors ^46– 49^. There is much debate over the pathogenesis of PRP: infectious, inflammatory, and vitamin A-associated etiologies have been proposed ^46, 50– 52^. A small fraction of PRP cases (less than 5%) are familial and are inherited in an autosomal dominant fashion ^46, 53, 54^.

In 2012, Fuchs-Telem *et al.* studied four unrelated families with familial PRP and identified three distinct mutations in caspase recruitment domain family member 14 ( *CARD14*) ^55^. *CARD14* is a known modulator of nuclear factor kappa B (NF-κB), which plays an important role in inflammatory pathways ^56, 57^. Fuchs-Telem *et al.* showed that NF-κB signaling is activated in PRP-affected skin and suggested that this inflammatory upregulation may play a role in the pathogenesis of familial PRP. Interestingly, causative mutations in *CARD14* were previously identified in familial psoriasis, and enhanced NF-κB signaling was also identified as a possible pathogenic factor ^58^. Taken together, these findings indicate that in addition to similarities in phenotypic features, familial PRP and familial psoriasis may share a common pathophysiology. Given the overall poor response to current treatments for PRP, this finding may allow for new therapeutic approaches that are aimed at modulating the immune response.

***Specific mutations in *TGM1* cause bathing-suit ichthyosis.*** As discussed above, ARCI encompasses a wide range of phenotypes, including LI, CIE, and HI. The most common underlying gene defect is the tranglutaminase-1 gene ( *TGM1*), which accounts for approximately 30% of heritability of ARCI ^17^.

Bathing-suit ichthyosis (BSI) is a rare variant of ARCI and is distinguished from the other forms of ARCI by restriction of scaling to the trunk, with sparing of the central face, buttocks, and limbs. All cases of BSI published to date have been caused by mutations in *TGM1*, although mutations in *TGM1* more commonly cause generalized ARCI ^59– 61^. In 2006, Oji *et al.* showed that the specific *TGM1* mutations that cause BSI may lead to temperature-dependent activity of transglutaminase, with marked decrease in enzyme function at higher temperatures ^61^. This may account for the differential scaling observed in BSI, with greater disease manifestation at sites with relatively higher temperature, such as the trunk. More recently, several studies have identified additional mutations in *TGM1* that contribute to BSI ^62– 64^, furthering the genetic understanding of this rare type of ichthyosis.

### Syndromic ichthyoses

***Mutations in *DSP* cause erythrokeratodermia-cardiomyopathy syndrome.*** In 2016, Boyden *et al.* identified a novel cardio-cutaneous disorder known as erythrokeratodermia-cardiomyopathy (EKC) syndrome. Early clinical findings include generalized erythrokeratodermia, recurrent infections, and failure to thrive as well as wiry or absent hair, dental enamel defects, absence of secondary teeth, and nail dystrophy. A hallmark of EKC is initially asymptomatic, rapidly progressive, potentially fatal cardiomyopathy, which was found in all three patients with EKC published in the literature to date ^65^.

EKC is caused by mutations in *DSP*, which encodes desmoplakin, a primary component of desmosomes ^65^. Desmosomes are intercellular adhesion junctions that are most abundant in the epidermis and the heart. *DSP* has been previously implicated in several disorders, including diseases with isolated cardiac manifestations and cardio-cutaneous syndromes. Examples of disorders caused by mutations in *DSP* include striate palmoplantar keratoderma, smooth palmoplantar keratoderma with woolly hair, Carvajal syndrome (dilated cardiomyopathy with woolly hair and keratoderma), and arrhythmogenic cardiomyopathy ^66– 70^. However, EKC syndrome represents a distinct clinical phenotype and is the only disorder caused by mutations in *DSP* to present with erythrokeratodermia ^65^.

Although EKC syndrome is a distinct entity based on specific clinical features and unique pathobiology, its initial clinical manifestation can appear very similar to CIE, which would not prompt any further cardiac evaluation. The description of this novel syndrome emphasizes the critical, potentially life-saving importance of genetic diagnosis in patients with ichthyosis.

***Mutations in *ELOVL4* cause ichthyosis, intellectual disability, and spastic quadriplegia.*** In 2011, Aldamesh *et al.* identified a novel neuro-ichthyotic disease caused by autosomal recessive mutations in *ELOVL4* and characterized by ichthyosis, mental retardation, seizures, and spastic quadriplegia ^71^. These phenotypic manifestations are similar to those of Sjögren-Larrson syndrome, a syndromic ichthyosis caused by mutations in fatty aldehyde dehydrogenase ( *ALDH3A2*) ^30^, but the neurologic findings in this newly described disorder are more severe ^71^.

*ELOVL4* is a fatty acid elongase and plays a crucial role in the synthetic pathway of very-long-chain fatty acids (VLCFAs) ^72^. VLCFAs have a wide range of functions, including cell signaling and maintenance of the epidermal barrier ^73– 78^. Heterozygous mutations in *ELOVL4* had been previously reported to cause macular degeneration ^79^, but Aldamesh *et al.* were first to report the involvement of homozygous *ELOVL4* mutations in an ichthyosis syndrome, with both cutaneous and neurologic findings. In addition to identifying a novel syndromic ichthyosis, this finding highlights the importance of VLCFAs in both brain development and maintenance of epidermal barrier function. It also suggests the use of VLCFA replacement therapy as a possible therapeutic option for the treatment of patients with this disorder.
